# Clinical management and outcomes for 51 Pugs reportedly diagnosed with protein‐losing enteropathy using VetCompass primary care veterinary data

**DOI:** 10.1111/jsap.70094

**Published:** 2026-01-25

**Authors:** A. Kathrani, D. C. Brodbelt, D. B. Church, D. G. O’Neill

**Affiliations:** ^1^ Department of Clinical Science and Services Royal Veterinary College Hatfield UK; ^2^ Department of Pathobiology and Population Sciences Royal Veterinary College Hatfield UK

## Abstract

**Objectives:**

To report the clinicopathological findings, treatment and outcomes of Pugs diagnosed with protein‐losing enteropathy using VetCompass primary care clinical data in the UK and to determine if referral or any specific treatments for protein‐losing enteropathy were associated with outcome.

**Materials and Methods:**

Retrospective VetCompass study of primary care practice records for Pugs with a recorded diagnosis of protein‐losing enteropathy. Of the 51 Pugs diagnosed with protein‐losing enteropathy between 2017 and 2024 in the UK, clinicopathological results, referral status, treatment and outcome on protein‐losing enteropathy were extracted.

**Results:**

Twenty‐one of 51 Pugs (41.2%) were referred for protein‐losing enteropathy care. Thirty‐four (66.7%) were treated with prednisolone, 2 (3.9%) with cyclosporin, 6 (11.8%) with chlorambucil and 14 (27.5%) with clopidogrel. Twenty‐two of 51 Pugs (43.1%) died presumed due to their protein‐losing enteropathy, with 54.5% of these deaths occurring within 3 months of diagnosis. More Pugs alive at 3 months compared to those that died within 3 months were treated with prednisolone (28/34 alive vs. 5/12 dead; *P*=.032) and clopidogrel (14/34 alive vs. 0/12 dead; *P*=.009). There was no difference between referred and non‐referred Pugs in their probability of being alive versus those that died due to their protein‐losing enteropathy at 3 months (17/34 alive vs. 3/12 dead; *P*=.183), 1 year (11/24 alive vs. 6/17 dead; *P*=.539) and 2 years (7/13 alive vs. 8/19 dead; *P*=.720).

**Clinical Significance:**

Studies that account for severity of disease are needed to determine if Pugs with protein‐losing enteropathy managed completely in primary care have similar outcomes versus referral. Prednisolone and clopidogrel may increase short‐term survival; however, prospective studies are needed to confirm this.

## INTRODUCTION

Dogs with protein‐losing enteropathy (PLE) due to chronic inflammatory enteropathy (CIE) and intestinal lymphangiectasia (IL) are reported in the literature with a guarded prognosis, with approximately 50% of referred cases being euthanised due to PLE (Craven & Washabau, 2019). Dogs with PLE due to either small‐ or large‐cell lymphoma are reported with significantly shorter survival times than PLE cases with CIE and IL (Nakashima et al., 2015). However, most studies assessing treatment response and outcome in dogs with PLE have focused only on referral caseloads (Allenspach et al., 2017; Dandrieux et al., 2013; Economu et al., 2021; Green & Kathrani, 2022; Hawes & Kathrani, 2024; Jablonski et al., 2024; Kathrani et al., 2019; Nakashima et al., 2015; Salavati Schmitz et al., 2019; Wootton et al., 2023), with these results likely being poorly generalisable to the wider population of PLE cases under primary veterinary care (Bartlett et al., 2010).

Predispositions and characteristics of PLE have been reported for different dog breeds in referral practice (Berghoff et al., 2007; Breitschwerdt, 1992; Littman et al., 2000; Ohmi et al., 2011; Simmerson et al., 2014). Basenjis are reported as predisposed to a type of PLE termed immunoproliferative enteropathy, with distinctive characteristics including hypergammaglobulinaemia (Breitschwerdt, 1992). Recently, Pugs with PLE were reported with distinctly poorer outcomes compared to other breeds clinically managed in referral practice (Hawes & Kathrani, 2024; Swales et al., 2025). Pugs diagnosed with PLE under referral care in the UK were 4.93 times more likely to die or be euthanised prior to hospital discharge compared to all other breeds, with 5/6 of these deaths in Pugs ascribed to presumptive aspiration pneumonia (Hawes & Kathrani, 2024). A more recent multi‐centre UK referral study of PLE similarly reported Pugs with higher proportional mortality compared to all other breeds with PLE (Swales et al., 2025). Both studies also reported that Pugs with PLE not surviving to hospital discharge had higher neutrophil counts and that neutrophil counts in peripheral blood were associated with an increased hazard of death, respectively (Hawes & Kathrani, 2024, Swales et al., 2025). However, the generalisability of these referral findings to the wider primary veterinary care population is uncertain and it remains unknown whether Pugs diagnosed with PLE and clinically managed only in primary care practice have similar outcomes to Pugs in referral practice.

The current study aimed to report the clinicopathological findings, treatment and outcome in Pugs diagnosed with PLE using the VetCompass primary care data, in order to capture a broader range of cases being diagnosed and managed either totally within primary care or also within referral level. A second aim was to characterise mortality in Pugs diagnosed with PLE using these data. A final aim was to determine if referral or any specific treatments for PLE were associated with outcome in Pugs with PLE.

## MATERIALS AND METHODS

### Study population

The study population included 40,509 Pugs under UK primary veterinary care during 2019 at clinics participating in the VetCompass Programme. Dogs under veterinary care were defined as those with either (a) at least one electronic health record (EHR) (free‐text clinical note, treatment, or bodyweight) recorded during 2019 or (b) at least one EHR recorded during both 2018 and 2020. VetCompass collates de‐identified EHR data from primary care veterinary practices in the UK for epidemiological research. Data fields available to VetCompass researchers include a unique animal identifier along with species, breed, date of birth, sex and neuter status, and clinical information from free‐form text clinical notes, bodyweight and treatment with relevant dates. Ethical approval was granted by the Social Science Research Ethical Review Board at the Royal Veterinary College (URN SR2024‐01632811).

### Case selection

A case series of Pugs diagnosed with PLE was undertaken within this VetCompass population. Case‐finding for dogs with a possible PLE diagnosis involved initial screening of all 40,509 Pugs by searching the clinical notes using the term “protein‐losing enteropathy” at any date (Paiva, 2013). This identified 77 candidate cases that were randomly ordered and had their clinical notes manually reviewed in detail to evaluate them for case inclusion. Case inclusion criteria required: 1. serum biochemistry confirmed panhypoproteinaemia without moderate or severe anaemia (moderate or severe anaemia defined as PCV or haematocrit <25%), nor evidence of blood loss to explain the panhypoproteinaemia and/or 2. the primary care practitioner recorded in the EHR that serum biochemistry results were consistent with PLE. In addition, cases meeting either of these two criteria also had to have PLE considered as the most likely diagnosis after clinical signs, physical examination findings, results of diagnostic investigations, response to treatment for PLE and follow‐up period were taken into consideration by one of the authors (board‐certified in small animal internal medicine). For candidate cases where only hypoalbuminaemia was present or mentioned, inclusion criteria required urinalysis with dipstick protein analysis or urine protein: creatinine ratio if concurrent hypocholesterolaemia was not present, basal cortisol or ACTH stimulation test if suspicion of hypoadrenocorticism was present on haematology (lack of stress leukogram) and/or abdominal imaging (small adrenal glands) and abdominal ultrasound or CT to rule out hepatic or other extra‐gastrointestinal causes. Exclusion criteria included insufficient medical records to meet inclusion criteria, as described above.

### Data collection

The EHRs from all dogs that met the inclusion criteria were reviewed in detail to extract the following information for each case: 1. Signalment; 2. clinical signs at diagnosis, with diagnosis defined as the time of serum biochemistry documenting panhypoproteinaemia or hypoalbuminaemia; 3. physical examination at diagnosis; 4. diagnostic investigations and results for PLE; 5. treatment at diagnosis; 6. treatment at follow‐up; 7. whether the case was referred; 8. whether the case was alive or dead at the final available EHR; 9. reason and timing of death if applicable; and 10. duration of follow‐up; defined as time from diagnosis until last entry in EHR.

### Statistical analysis

Statistical analyses were performed using a computer software package (IBM SPSS Statistics Version 29). Normality was assessed for continuous variables using the Shapiro–Wilk test. Results were reported as mean ± standard deviation [SD] if normally distributed and as median with interquartile range [IQR] and range if non‐normally distributed. For categorical data, results were described as numbers and percentages of cases affected.

Variables included for statistical analysis of outcome at 3 months, 1 year and 2 years were as follows: 1. whether the case was referred and 2. treatment (oral or parenteral cobalamin, prednisolone <7.5 mg/day or 7.5 mg/day or above, diet [low‐fat, hydrolysed protein, gastrointestinal, home‐cooked, other], cyclosporin, chlorambucil and clopidogrel). Outcome was grouped according to numbers documented to be alive or dead due to PLE at 3 months, 1 year and 2 years from diagnosis. Chi‐squared or Fischer’s exact testing was used to identify significant associations between categorical variables. Significance was defined as *P* < .05. Kaplan–Meier estimates of survival based on death due to PLE in Pugs were calculated using IBM SPSS Statistics Version 29.

## RESULTS

### Study dogs

#### Inclusion criteria

Of the 77 candidate PLE cases identified in the searching, 26 (33.8%) Pugs had insufficient medical records to meet inclusion criteria and were therefore excluded from analysis. This left 51 Pugs (66.2%) who met the PLE inclusion criteria. Of these, serum biochemistry confirmed panhypoproteinaemia without moderate or severe anaemia (PCV or haematocrit <25%) nor evidence of blood loss in 25 dogs (49.0%) and the primary care practitioner recorded in the EHR that serum biochemistry results were consistent with PLE for 6 dogs (11.8%). Of the 25 dogs included due to panhypoproteinaemia, 20 presented with diarrhoea, 3 with bloating or ascites, 2 with vomiting and 1 with weight loss. Collectively, the 31 dogs with panhypoproteinaemia or biochemistry results reported to be consistent with PLE had the following diagnostic investigations reported for their PLE: serum biochemistry (31, 100%), haematology (31, 100%), urinalysis (10, 32.3%), faecal analysis (12, 38.7%), serum cobalamin (13, 41.9%), basal cortisol or ACTH stimulation test (6, 19.4%), pre‐prandial or bile acid stimulation test (2, 6.5%), trypsin like immunoreactivity (6, 19.4%), pancreatic lipase immunoreactivity (5, 16.1%), gastrointestinal endoscopy (5, 16.1%), exploratory laparotomy (3, 9.7%) and intestinal biopsies (6, 19.4%; 5 *via* endoscopy, 1 *via* exploratory laparotomy). For the 20 (39.2%) PLE cases where only hypoalbuminaemia was present or mentioned, all dogs had abdominal imaging (19 abdominal ultrasound, 1 abdominal CT), 4 had low serum cholesterol and for the 16 dogs with no mention of low serum cholesterol, all had urine protein analysis *via* dipstick or urine protein: creatinine ratio. Six dogs had basal cortisol or ACTH stimulation test; no dog without this test had absence of a stress leukogram. Additional tests performed for these 20 dogs included: faecal analysis (9, 45%), serum cobalamin (12, 60%), trypsin like immunoreactivity (6, 30%), pancreatic lipase immunoreactivity (2, 10%), pre‐prandial bile acids or bile acid stimulation test (4, 20%), gastrointestinal endoscopy (2, 10%) and gastrointestinal biopsies (3, 15%; 2 *via* endoscopy, 1 unclear method of collection).

#### Clinical signs and duration at diagnosis

Of the 51 Pugs with PLE included in the study, median age at first diagnosis was 96 months (range 21 to 168) and there were 7 male entire, 17 male neutered, 6 female entire and 21 female neutered dogs. At diagnosis, 37/51 (72.5%) PLE cases were reported with some form of diarrhoea (21 (41.2%) unspecified diarrhoea, 10 (19.6%) watery/liquid diarrhoea, 4 (7.8%) diarrhoea with mucus/fresh blood, 2 (3.9%) water/liquid diarrhoea with mucus/fresh blood) and 1 (2.0%) dog was specifically reported not to have diarrhoea. Additional clinical signs reported at diagnosis included 17 (33.3%) with vomiting, 16 (31.4%) with inappetence, 10 (19.6%) with lethargy, 9 (17.6%) with weight loss, 13 (25.5%) with increased drinking, 8 (15.7%) with flatulence and 5 (9.8%) with bloating. Thirteen dogs (25.5%) had clinical signs associated with their PLE for more than 3 weeks prior to diagnosis.

#### Physical examination findings at diagnosis

During physical examination at diagnosis, 16 dogs (31.4%) were reported to have bloated/distended abdomen, ascites or peripheral oedema on physical examination and 3 (5.9%) had pale mucous membranes.

#### Clinicolaboratory variables and abdominal ultrasound

At diagnosis, serum albumin concentrations were reported to be below 15 g/L in 15 dogs (29.4%) and above 15 g/L in 14 (27.5%). Haematocrit or packed cell volume (PCV) was reported to be mildly–moderately decreased (defined as a haematocrit or PCV > 25%) in 19 dogs (37.3%). Seven dogs (13.7%) had increased platelets and 22 dogs (43.1%) had changes on their leukogram, defined as neutrophilia and/or monocytosis. Serum potassium concentration was increased in 3 dogs (5.9%) and for 6 (11.8%) the sodium: potassium ratio was reported to be decreased. Nineteen dogs (37.3%) had serum cobalamin concentrations below the reference interval, serum folate was decreased in 6 (11.8%) and increased in 5 (9.8%). Six dogs (11.8%) had hyposthenuria, and 9 (17.6%) had changes in the jejunum on abdominal ultrasound (5 with wall thickening, 3 with mucosal speckles or striations, 1 with loss of wall layering).

#### Endoscopy, exploratory laparotomy and intestinal histopathology

Seven dogs (13.7%) underwent endoscopy and 3 (5.9%) had exploratory laparotomy. Nine (17.6%) had intestinal histopathology performed and all were consistent with chronic inflammatory enteropathy, with one also having concurrent lymphangiectasia.

### Referral for PLE

Twenty‐one (41.2%) of the PLE cases were also seen under referral care related to their PLE condition.

### Treatment of PLE at diagnosis

Treatment included oral or parenteral cobalamin supplementation in 24 dogs (47.1%), prednisolone in 34 dogs (66.7%; <7.5mg/day for 10 dogs and 7.5mg/day or above for 24 dogs), diet [low‐fat 5 (9.8%), hydrolysed protein 22 (43.1%), gastrointestinal 11 (21.6%), home‐cooked 1 (2.0%), other 1 (2.0%)], cyclosporin in 2 (3.9%), chlorambucil in 6 (11.8%) and clopidogrel in 14 (27.5%). Of the 14 dogs that received clopidogrel at diagnosis, 13 also received prednisolone.

### Outcome

Twenty‐five (49.0%) Pugs with PLE were alive at the end of the study with a median follow‐up of 17 months [interquartile range (IQR) 3.5 to 30 months, range 1 day to 57 months]. Twenty (80.0%) of these 25 Pugs were documented to be alive at 3 months (median follow‐up: 19.5 months, range 3 to 57 months), 18 (72.0%) at 1 year (median follow‐up: 22 months, range 12 to 57 months) and 9 (36.0%) at 2 years (median 36 months, range 24 to 57 months). Seven Pugs (28.0%) were alive for 2 years or more (median follow‐up 38 months, range 28 to 57 months).

Overall, 22 Pugs (43.1%) died due to PLE during the study period. Of these, 12 (54.5%) died within 3 months of diagnosis (median 22 days, IQR 6 days to 2 months, range 1 day to 2 months), 17 (77.3%) within one year of diagnosis (median 2 months, IQR 10 days to 4 months, range 1 day to 9 months) and 19 (86.4%) within 2 years of diagnosis (median 2 months, IQR 11 days to 5 months, range 1 day to 22 months). Three Pugs (13.6%) died presumed from PLE after 2 years of diagnosis (34, 44 and 46 months after diagnosis).

**FIG 1:**
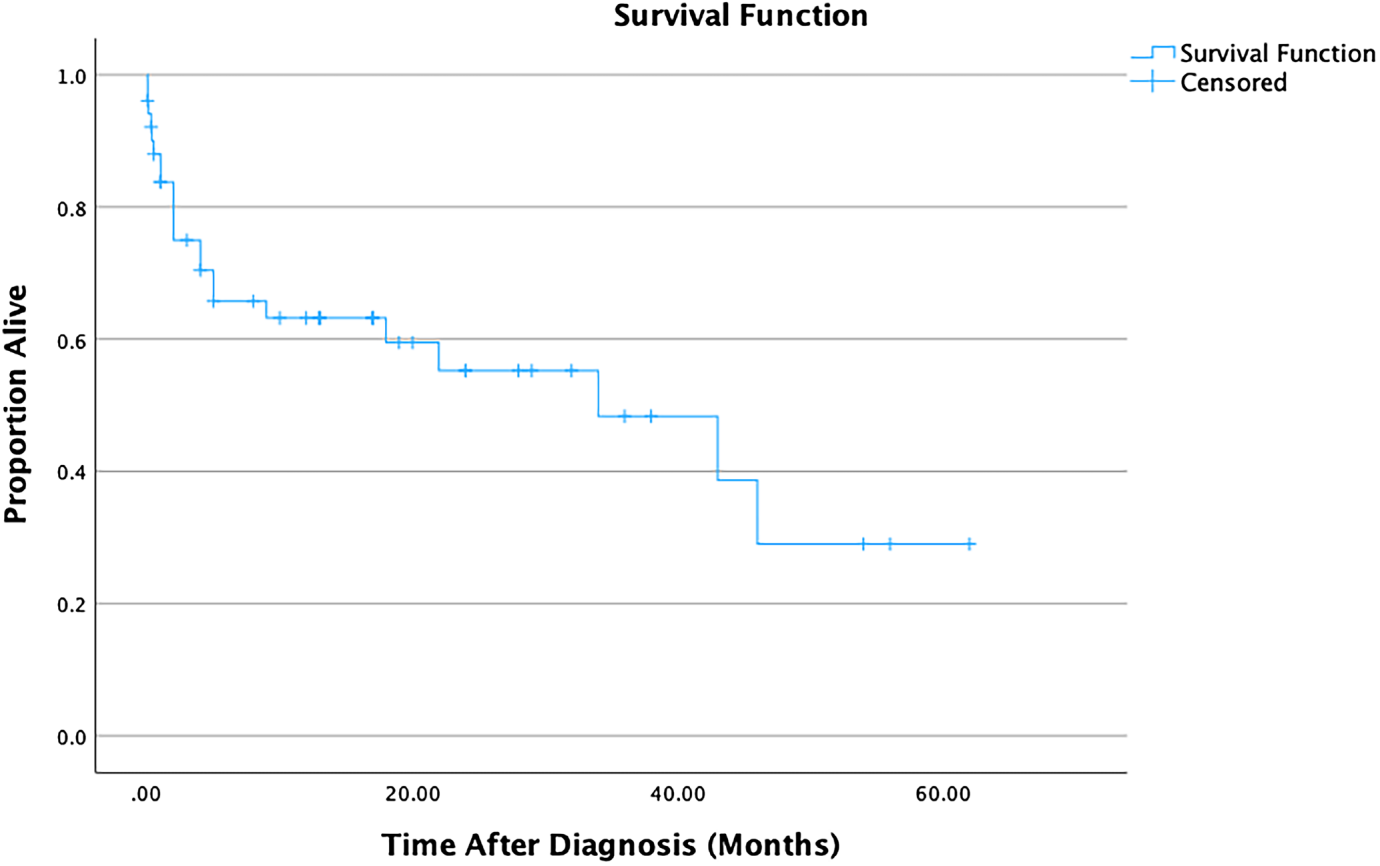
Kaplan–Meier estimates of survival based on death due to protein‐losing enteropathy in Pugs. Marks in the lines indicate censored dogs, which were defined as dogs alive at the time of follow‐up (*n* = 25) or dead due to other or unknown causes (*n* = 4).

Of the 4 Pugs that died during the study but unrelated to their PLE, 3 Pugs (75%) died due to unknown cause (8 months, 10 months and 2 years and 5 months after diagnosis) and 1 (25%) died due to urethral carcinoma (5 months after diagnosis). Kaplan–Meier estimates of survival based on death due to PLE in Pugs are depicted in Fig. 1.

The probability of being alive or dead due to PLE at the end of the study period was not statistically significantly associated with whether the Pug was referred (*n* = 18) or not referred (*n* = 29) for clinical management of PLE: alive due to PLE at end of study (*n* = 25): referred 9/25, not referred 16/25 versus dead due to PLE at end of study (*n* = 22): referred 9/22, not referred 13/22; (*P* = .771).

### Causes and signs at the time of death or euthanasia

Fourteen of the 22 (63.6%) Pugs who died related to PLE were euthanised and 8 (36.4%) had an unassisted death. Clinical findings recorded at the time of euthanasia or unassisted death in these 22 dogs included: vomiting [6 (27.3%)], reduced appetite (5; 22.7%), diarrhoea [3 (13.6%), 1 with melaena], aspiration pneumonia (1; 4.5%), laboured breathing (2; 9.1%), lethargy and/or weakness (10; 45.5%), seizures [3 (13.6%), none reported prior to diagnosis], collapse (4; 18.2%), trembling/shaking (3; 13.6%), vacant (1; 4.5%), pale mucous membranes (3; 13.6%), anaemia (4; 18.2%), sepsis (1; 4.5%), pancytopaenia (1; 4.5%), foam from nose following dead on arrival (1; 4.5%).

Of the 6 dogs that received chlorambucil at diagnosis, one was present in the 22 dogs that died related to PLE, and this dog had pancytopaenia documented at the time of death. Of the 14 dogs that received clopidogrel at diagnosis, 3 were present in the 22 dogs that died related to PLE. Of these 3 dogs, one had aspiration pneumonia, one collapse and melaena and 1 was lethargic and weak with pale mucous membranes and seizures.

### Statistical analysis

#### Three‐month outcome

Twelve Pugs had died due to PLE within 3 months. Thirty‐four Pugs were documented to still be alive at 3 months follow‐up. Five Pugs did not have enough follow‐up to determine outcome at 3 months and were therefore excluded from statistical analysis (median follow up: 15 days following diagnosis, range: 1 day to 1 month) (Table 1).

**Table 1:** Statistical analysis using chi‐squared or Fischer’s exact testing of referral status and treatment in 51 Pugs with protein‐losing enteropathy (PLE) using VetCompass primary care data in the UK and respective outcomes

Variables	3 months			1 year			2 years		
	Alive (34)	Dead due to PLE (12)	*P*	Alive (24)	Dead due to PLE (17)	*P*	Alive (13)	Dead due to PLE (19)	*P*
Referred	17	3	.183	11	6	.539	7	8	.720
Treatment: B12	17	5	.742	11	7	1.000	4	9	.471
Treatment: prednisolone									
<7.5 mg/day	8	2	.**032**	6	3	.154	4	3	.087
≥7.5 mg/day	20	3		14	6		3	8	
Treatment: diet									
Low fat	5	0	.538	4	0	.387	3	0	.077
HA	16	5		11	9		2	11	
GI	6	3		3	4		3	4	
Home‐cooked	0	0		0	0		0	0	
Other	1	0		1	0		0	0	
Treatment: cyclosporine	2	0	1.000	2	0	.502	1	1	1.000
Treatment: chlorambucil	6	0	.317	5	1	.373	4	1	.132
Treatment: clopidogrel	14	0	.**009**	9	2	.085	5	3	.219

Significant *P* value (<.05) is listed in bold

HA Hydrolysed protein, GI Gastrointestinal

Of the treatment variables tested (Table 1), the following differed significantly between dogs that had died due to PLE (*n* = 12) or were documented to still be alive at 3 months follow‐up (*n* = 34): prednisolone at diagnosis (alive at 3 months: no prednisolone [6/34], prednisolone at <7.5 mg/day [8/34], prednisolone at 7.5 mg/day or above [20/34] vs. dead within 3 months: no prednisolone [7/12], prednisolone at <7.5 mg/day [2/12], prednisolone at 7.5 mg/day or above [3/12]; *P*=.032) and clopidogrel at diagnosis: (alive at 3 months: no clopidogrel [20/34], clopidogrel [14/34] vs. dead within 3 months: no clopidogrel [12/12], clopidogrel [0/12]; *P*=.009) (Table 1). The one dog that received clopidogrel without prednisolone at diagnosis was alive at 3 months (euthanised at 8 months for unknown cause).

There was no significant association between being referred and the outcome at 3 months: alive at 3 months (17/34 referred, 17/34 non‐referred) versus dead within 3 months (3/12 referred, 9/12 non‐referred), *P*=.183.

#### One‐year outcome

Seventeen Pugs had died due to PLE within 1 year. Twenty‐four Pugs were documented to still be alive at 1‐year follow‐up. Three Pugs died of causes other than PLE (1 urethra carcinoma at 5 months and 2 due to unknown cause at 8 months and 10 months). Seven Pugs did not have enough follow‐up to determine outcome at 1 year (median follow up: 1 month following diagnosis, range: 1 day to 4 months). Dogs that died of other causes and did not have follow‐up 1 year following diagnosis were excluded from statistical analysis, Table 1. None of the treatment variables tested showed a statistically significant association with an outcome of either having died due to PLE (*n* = 17) or were documented to still be alive at 1 year follow‐up (*n* = 24) (*P*>.085, Table 1). There was no significant association between being referred and the outcome at 1 year: alive at 1 year (11/24 referred, 13/24 non‐referred) versus dead within 1 year (6/17 referred, 11/17 non‐referred), *P*=.539.

#### Two‐year outcome

Nineteen Pugs had died due to PLE within 2 years. Thirteen Pugs were documented to still be alive at 2 years follow‐up. Three Pugs died of causes other than PLE (1 urethra carcinoma at 5 months and 2 due to unknown cause at 8 months and 10 months). Sixteen Pugs did not have enough follow‐up to determine outcome at 2 years (median follow up: 12.5 months following diagnosis, range: 1 day to 20 months). Dogs that died of other causes and did not have follow‐up 2 years following diagnosis were excluded from statistical analysis (Table 1). None of the treatment variables tested showed a statistically significant association with the outcome of either having died due to PLE (*n* = 19) or were documented to still be alive at 2 years follow‐up (*n* = 13) (*P*>.077, Table 1). There was no significant association between being referred and the outcome at 2 years: alive at 2 years (7/13 referred, 6/13 non‐referred) versus dead within 2 years (8/19 referred, 11/19 non‐referred), *P*=.720.

## DISCUSSION

The current study follows a case series of Pugs reported to be diagnosed with PLE using VetCompass primary care data to report the clinicopathological findings, treatment and outcome, as well as characterise mortality and whether referral or any specific treatments of PLE were associated with outcome. Of the clinical signs reported at diagnosis, increased drinking (verified in some dogs by hyposthenuria), flatulence, bloating and vomiting were apparent in some Pugs with PLE. Comparison of these clinical signs to other breeds predisposed to PLE will help to determine how unique these disease characteristics are for the Pug breed. Also, clinicopathological abnormalities such as anaemia, thrombocytosis, neutrophilia, monocytosis, hyperkalaemia and decreased sodium: potassium ratio were reported in the current PLE cases, as well as changes in the jejunum on abdominal ultrasound. There are many underlying causes for PLE, such as CIE, IL and gastrointestinal lymphoma (Craven & Washabau, 2019), and therefore, clinical signs and clinicolaboratory findings are likely to vary with the different underlying disease profiles across breeds. Unfortunately, the underlying cause for the PLE diagnosis in most of the Pugs in our study was not identified, as most (82.4%) did not have gastrointestinal histopathology performed. Therefore, more research comparing different clinical features of PLE, as well as the underlying cause in various breeds, will help to highlight further breed‐specific disease characteristics, which may then help to elucidate breed‐specific pathogenesis that would allow tailoring of treatment to try to improve outcome.

The current study reported an overall mortality rate due to PLE of 43.1%, which is similar to previous studies reporting mortality rate in dogs with PLE (Allenspach et al., 2017; Craven & Washabau, 2019; Kathrani et al., 2019). However, previous studies reporting mortality rate have assessed this in a referral population of dogs with PLE and have typically used relatively shorter follow‐up times compared to our study. Therefore, our study focused on Pugs with PLE diagnosed under primary care and then treated at either or both primary care practice only (58.8%) or primary care and referral practices (41.2%) and reported mortality due to PLE at several time points, including up to 2 years follow‐up. Interestingly, our study found no significant difference in outcome between those Pugs that were referred for their PLE versus those PLE cases that were managed entirely under primary care. This finding may reflect the severity of PLE in Pugs with a generally poor prognosis and therefore outcome may be independent of referral. This is highlighted by the result in our study that just over one‐third of Pugs that died due to PLE (36.4%) had an unassisted death rather than being euthanised. Therefore, further research investigating the pathogenesis and optimal treatment of PLE in Pugs is needed to improve the prognosis. At all three time points of 3 months, 1 year and 2 years, there was no statistically significant difference in survival between Pugs with PLE that had been referred compared to unreferred PLE cases. However, the current study included only 51 PLE cases and therefore this failure to detect differences may have been a Type II error where a false‐negative result may have been because the study was underpowered. Therefore, further studies using larger sample sizes are needed for greater certainty on the impact of referral on the outcome of this disease. Furthermore, the severity of disease should also be taken into consideration for future studies assessing the impact of referral, as the lack of significance in our study could have also been due to milder cases being treated in primary care resulting in better outcomes versus more severe cases being referred, which could have resulted in worse outcomes despite referral, due to the nature of their disease severity.

Causes of death or clinical signs at the time of death or euthanasia were varied and only included one case of aspiration pneumonia, which is a lower frequency compared to previous reports in the subset of Pugs with PLE in referral practice (Hawes & Kathrani, 2024; Swales et al., 2025). However, this low proportion of aspiration pneumonia in the current study could have been due to decreased awareness by clinicians for this potential complication or lack of expertise or equipment to diagnose aspiration pneumonia in primary care practices. Interestingly, our study showed one of the most common signs recorded at the time of death or euthanasia included vomiting (27.3%), which could predispose to aspiration events. Therefore, owners of dogs with PLE should be warned to monitor for vomiting during the course of disease so that timely treatment can be sought that may decrease the risk of aspiration pneumonia. Although vomiting was recorded in 33.3% of the Pugs with PLE at presentation in the current study, this proportion was similar to a previous referral study including 107 dogs with PLE consisting of various breeds at 31.8% (Hawes & Kathrani, 2024). Therefore, whether Pugs with PLE have increased susceptibility to aspiration pneumonia and whether this is due to the nature or frequency of vomiting, or another reason, such as their brachycephalic conformation or oesophageal function remains to be answered.

Other commonly reported clinical signs at the time of death or euthanasia included pale mucous membranes (13.6%), seizures (13.6%), and anaemia (18.2%). None of the dogs that exhibited these specific signs had received chlorambucil at diagnosis. These findings have not previously been reported in the literature and are therefore worthy of further discussion and future investigation. Pale mucous membranes could reflect anaemia or hypovolaemia (Kisielewicz et al., 2014). Possible causes for anaemia in dogs with PLE include anaemia of chronic disease, which is typically mild and non‐regenerative or anaemia due to gastrointestinal blood loss, which could be regenerative (Chikazawa & Dunning, 2016). Unfortunately, further detail regarding the regenerative nature of the anaemia in the Pugs in the current study was unavailable to help ascertain a likely cause. Therefore, this should be investigated in the future to help determine if gastrointestinal bleeding is associated or a complication of PLE in Pugs and whether addressing this could help to improve survival. Interestingly, three Pugs with PLE that died or were euthanised in the current study were recorded with seizures at the time of death that were new in onset. Seizure has been reported in a dog with PLE previously, due to hypocalcaemia presumed due to hypovitaminosis D (Whitehead et al., 2015). Alternatively, thromboembolic events have also been a complication of PLE (Jacinto et al., 2017; Oishi et al., 2025) and if occurring in the brain could result in a vestibular event which could be confused as a seizure by the owner. Further awareness and better characterisation of these reported seizure episodes by veterinary surgeons and owners will help determine their exact nature so specific treatment can be initiated, which may help to improve outcome. For example, if the seizure episodes are linked to hypovitaminosis D or a thromboembolic event, treatment aimed at correcting vitamin D status, or medications reducing hypercoagulability can then be used to improve prognosis.

Pugs with PLE treated with prednisolone were significantly more likely to be alive at 3 months, but these treatment effects were no longer significant at the 1‐year or 2‐year outcome analysis. The finding that prednisolone may improve short‐term survival is not surprising given that one of the causes of PLE is a chronic enteropathy. Chronic enteropathy can be associated with marked inflammation and therefore treatment with prednisolone may help to reduce the inflammation and improve outcome (Allenspach et al., 2007). One study in dogs with PLE showed that those that were immunosuppressant‐responsive or nonresponsive versus food‐responsive had a canine chronic enteropathy clinical activity index (CCECAI) score of higher than 8 (Nagata et al., 2020). Similarly, another study showed that the increased CCECAI at the time of diagnosis was associated with the outcome of death in dogs with PLE (Kathrani et al., 2019). Unfortunately, due to the retrospective nature of our study, we were unable to assign CCECAI scores to our Pugs with PLE. Therefore, we were unable to determine if CCECAI scores in our study correlated with death in Pugs with PLE and whether those with higher scores were more likely to respond to prednisolone use in the short‐term. In addition, our study assessed two doses of prednisolone: below 7.5 mg/day and 7.5 mg/day or above. Unfortunately, due to information on body weight not being consistently available in the record, we were unable to determine the mg/kg of prednisolone used in our study. Therefore, further studies are needed to determine the effect of CCECAI, as well as other variables at defining the subset sof Pugs that are most likely to benefit from prednisolone, as well as the optimum dose needed to improve outcome.

Pugs with PLE treated with clopidogrel were significantly more likely to be alive at 3 months, but these treatment effects were no longer significant at the 1‐year or 2‐year outcome analysis. Dogs with PLE are reportedly prone to thromboembolic events, due to loss of antithrombin and general increase in procoagulant state (deLaforcade et al., 2022; Goodwin et al., 2011; Wennogle et al., 2021). Given the results of our study with regards to clopidogrel use being zero in the death group at 3 months, this may highlight some potential value from clopidogrel medication in Pugs with PLE, presumably to reduce the frequency of thromboembolic events, which could lead to exacerbation of their signs or non‐response to treatment and ultimate death and euthanasia. Interestingly, some Pugs with PLE in our study and in another study have suggested thromboembolic events could be the cause for their deterioration or death, such as laboured breathing, sudden onset collapse, cardiopulmonary arrest and seizure‐like episodes, which could be due to ischaemic events (Swales et al., 2025). Therefore, future studies should prioritise confirming if clopidogrel helps increase survival, as well as defining which Pugs with PLE are to gain the greatest benefit from this treatment at diagnosis, as this may help improve outcome in this breed.

Our study had some important limitations. The use of retrospective clinical records that were not originally recorded for research purposes meant that medical records were not always complete. Therefore, some true clinical signs and physical examination findings may not have been entered and therefore were missing rather than truly not being present. Our inclusion criteria for PLE due to panhypoproteinaemia was based on the most likely diagnosis after reviewing the medical records and taking into consideration the history, presenting clinical signs, physical examination findings, results from diagnostic investigations, response to treatment for PLE and follow‐up period by one board‐certified small animal internal medicine specialist rather than two or more reaching a consensus, which would have been more robust. Correction for multiple testing was not pursued, and therefore, there is an increased risk of Type I error for our study. Consequently, the specific results for each individual test should be considered as hypothesis generating rather than confirmatory (Ranstam, 2019). Our study design also had elements of survival bias towards including milder cases, as only the subset of Pugs diagnosed before 2019 with PLE that survived into 2019 were included in the denominator study population (Smit et al., 2019). The underlying histopathological cause for the PLE was not determined for most of the cases in our study (82.4%), and furthermore, as the original reports were unavailable, it was unclear if the histopathology was reported according to the WSAVA Gastrointestinal Standardization Group guidelines or by a board‐certified veterinary pathologist. The cases included may not be a random sample of all PLE cases diagnosed in this population of dogs because the study design included only one search (protein‐losing enteropathy). The aim of the study was to identify a case series of PLE cases rather than to identify all cases diagnosed in the study cohort. In addition, cases that did not have enough follow‐up to the different time points could not be included in statistical analysis, and therefore, this could have resulted in selection bias. Finally, our statistical analysis was not adjusted for major confounders, and therefore, our results should be considered as hypothesis generating rather than confirmatory. However, we feel that our study is a first necessary step to further investigate the characteristics and outcome of Pugs with PLE presenting to both primary care and referral practices and forms a foundation to investigate further areas such as the onset of neurological signs following treatment, whether gastrointestinal bleeding occurs or increased thrombotic events after treatment and to determine if prednisolone or clopidogrel would improve the outcome of PLE in this breed. Also, utilising the large data set from primary care practices allows for many cases to be examined compared to solely referral practice records, and therefore, helps to increase the power of the study and ultimate comparisons between predisposed breeds, so unique breed characteristics and outcome can be determined to allow tailoring of breed specific treatment.

In conclusion, Pugs diagnosed with PLE in primary care practices had similar outcomes to those reported from referral practices. However, studies that account for the severity of disease are needed to determine if Pugs with PLE managed completely in primary care have similar outcomes versus referral. Prednisolone and clopidogrel treatment may improve short‐term outcomes. However, further studies will help elucidate the pathogenesis of PLE in Pugs, as well as optimum treatment to improve outcomes.

## Author contributions

**A. Kathrani:** hypothesis generation and experimental design, optimising and conducting experiments, interpreting and analysing the results, writing and revising the manuscript. **D. C. Brodbelt:** optimising and conducting experiments, interpreting and analysing the results, revising the manuscript. **D. B. Church:** optimising and conducting experiments, revising the manuscript. **D. G. O'Neill:** hypothesis generation and experimental design, optimising and conducting experiments, interpreting and analysing the results, revising the manuscript.

## Conflict of interest

The authors declare no potential conflicts of interest with respect to the research, authorship, and/or publication of this article. Dr Kathrani is an Associate Editor of the Journal of Small Animal Practice and co‐author of this article. She was excluded from editorial decision‐making related to the acceptance of this article for publication in the journal.

## Funding

The authors received no financial support for the research, authorship and/or publication of this article.

## Data Availability

The data that support the findings of this study are available from the corresponding author upon reasonable request.

## References

[jsap70094-bib-0001] Allenspach, K. , Rizzo, J. , Jergens, A.E. & Chang, Y.M. (2017) Hypovitaminosis D is associated with negative outcome in dogs with protein losing enteropathy: a retrospective study of 43 cases. BMC Veterinary Research, 13, 96. 28390394 10.1186/s12917-017-1022-7 PMC5385077

[jsap70094-bib-0002] Allenspach, K. , Wieland, B. , Grone, A. & Gaschen, F. (2007) Chronic enteropathies in dogs: evaluation of risk factors for negative outcome. Journal of Veterinary Internal Medicine, 21, 700–708. 17708389 10.1892/0891-6640(2007)21[700:ceideo]2.0.co;2

[jsap70094-bib-0003] Bartlett, P.C. , Van Buren, J.W. , Neterer, M. & Zhou, C. (2010) Disease surveillance and referral bias in the veterinary medical database. Preventive Veterinary Medicine, 94, 264–271. 20129684 10.1016/j.prevetmed.2010.01.007

[jsap70094-bib-0004] Berghoff, N. , Ruaux, C.G. , Steiner, J.M. & Williams, D.A. (2007) Gastroenteropathy in Norwegian Lundehunds. Compendium on Continuing Education for Veterinarians, 29, 456–465 468–470; quiz 470‐451. 17849700

[jsap70094-bib-0005] Breitschwerdt, E.B. (1992) Immunoproliferative enteropathy of basenjis. Seminars in Veterinary Medicine and Surgery (Small Animal), 7, 153–161. 1626155

[jsap70094-bib-0006] Chikazawa, S. & Dunning, M.D. (2016) A review of anaemia of inflammatory disease in dogs and cats. The Journal of Small Animal Practice, 57, 348–353. 27385622 10.1111/jsap.12498

[jsap70094-bib-0007] Craven, M.D. & Washabau, R.J. (2019) Comparative pathophysiology and management of protein‐losing enteropathy. Journal of Veterinary Internal Medicine, 33, 383–402. 30762910 10.1111/jvim.15406 PMC6430879

[jsap70094-bib-0008] Dandrieux, J.R. , Noble, P.J. , Scase, T.J. , Cripps, P.J. & German, A.J. (2013) Comparison of a chlorambucil‐prednisolone combination with an azathioprine‐prednisolone combination for treatment of chronic enteropathy with concurrent protein‐losing enteropathy in dogs: 27 cases (2007‐2010). Journal of the American Veterinary Medical Association, 242, 1705–1714. 23725434 10.2460/javma.242.12.1705

[jsap70094-bib-0009] deLaforcade, A. , Bacek, L. , Blais, M.C. , Boyd, C. , Brainard, B.M. , Chan, D.L. et al. (2022) 2022 update of the consensus on the rational use of Antithrombotics and thrombolytics in veterinary critical care (CURATIVE) domain 1 – defining populations at risk. Journal of Veterinary Emergency and Critical Care (San Antonio), 32, 289–314. 10.1111/vec.13204 PMC9322658 35499966

[jsap70094-bib-0010] Economu, L. , Chang, Y.M. , Priestnall, S.L. & Kathrani, A. (2021) The effect of assisted enteral feeding on treatment outcome in dogs with inflammatory protein‐losing enteropathy. Journal of Veterinary Internal Medicine, 35, 1297–1305. 33931908 10.1111/jvim.16125 PMC8163126

[jsap70094-bib-0011] Goodwin, L.V. , Goggs, R. , Chan, D.L. & Allenspach, K. (2011) Hypercoagulability in dogs with protein‐losing enteropathy. Journal of Veterinary Internal Medicine, 25, 273–277. 21314726 10.1111/j.1939-1676.2011.0683.x

[jsap70094-bib-0012] Green, J. & Kathrani, A. (2022) Incidence of relapse of inflammatory protein‐losing enteropathy in dogs and associated risk factors. Journal of Veterinary Internal Medicine, 36, 1981–1988. 36207819 10.1111/jvim.16561 PMC9708386

[jsap70094-bib-0013] Hawes, C. & Kathrani, A. (2024) In‐hospital mortality in dogs with protein‐losing enteropathy and associated risk factors. Journal of Veterinary Internal Medicine, 38, 2265–2272. 38819636 10.1111/jvim.17123 PMC11256150

[jsap70094-bib-0014] Jablonski, S.A. , Mazepa, A.S.W. & Tolbert, M.K. (2024) Use of octreotide for the treatment of protein‐losing enteropathy in dogs: retrospective study of 18 cases. Journal of Veterinary Internal Medicine, 38, 145–151. 38038236 10.1111/jvim.16966 PMC10800202

[jsap70094-bib-0015] Jacinto, A.M.L. , Ridyard, A.E. , Aroch, I. , Watson, P.J. , Morrison, L.R. , Chandler, M.L. et al. (2017) Thromboembolism in dogs with protein‐losing enteropathy with non‐neoplastic chronic small intestinal disease. Journal of the American Animal Hospital Association, 53, 185–192. 27841681 10.5326/JAAHA-MS-6328

[jsap70094-bib-0016] Kathrani, A. , Sanchez‐Vizcaino, F. & Hall, E.J. (2019) Association of chronic enteropathy activity index, blood urea concentration, and risk of death in dogs with protein‐losing enteropathy. Journal of Veterinary Internal Medicine, 33, 536–543. 30784115 10.1111/jvim.15448 PMC6430906

[jsap70094-bib-0017] Kisielewicz, C. , Self, I. & Bell, R. (2014) Assessment of clinical and laboratory variables as a guide to packed red blood cell transfusion of euvolemic anemic dogs. Journal of Veterinary Internal Medicine, 28, 576–582. 24417587 10.1111/jvim.12280 PMC4857975

[jsap70094-bib-0018] Littman, M.P. , Dambach, D.M. , Vaden, S.L. & Giger, U. (2000) Familial protein‐losing enteropathy and protein‐losing nephropathy in Soft Coated Wheaten Terriers: 222 cases (1983‐1997). Journal of Veterinary Internal Medicine, 14, 68–80. 10668820 10.1892/0891-6640(2000)014<0068:fpleap>2.3.co;2

[jsap70094-bib-0019] Nagata, N. , Ohta, H. , Yokoyama, N. , Teoh, Y.B. , Nisa, K. , Sasaki, N. et al. (2020) Clinical characteristics of dogs with food‐responsive protein‐losing enteropathy. Journal of Veterinary Internal Medicine, 34, 659–668. 32060974 10.1111/jvim.15720 PMC7096654

[jsap70094-bib-0020] Nakashima, K. , Hiyoshi, S. , Ohno, K. , Uchida, K. , Goto‐Koshino, Y. , Maeda, S. et al. (2015) Prognostic factors in dogs with protein‐losing enteropathy. Veterinary Journal, 205, 28–32. 26025135 10.1016/j.tvjl.2015.05.001

[jsap70094-bib-0021] Ohmi, A. , Ohno, K. , Uchida, K. , Nakayama, H. , Koshino‐Goto, Y. , Fukushima, K. et al. (2011) A retrospective study in 21 Shiba dogs with chronic enteropathy. The Journal of Veterinary Medical Science, 73, 1–5. 20716863 10.1292/jvms.10-0154

[jsap70094-bib-0022] Oishi, N. , Ohta, H. , Tamura, M. , Hanazono, K. , Miyoshi, K. , Yokoyama, N. et al. (2025) Prospective estimation of the prevalence of thromboembolism in dogs with inflammatory protein‐losing enteropathy. Journal of Veterinary Internal Medicine, 39, e70098. 40220264 10.1111/jvim.70098 PMC11992960

[jsap70094-bib-0023] Paiva, S. (2013) A fuzzy algorithm for optimizing semantic documental searched. Procedia Technology, 9, 1–10.

[jsap70094-bib-0024] Ranstam, J. (2019) Hypothesis‐generating and confirmatory studies, Bonferroni correction, and pre‐specification of trial endpoints. Acta Orthopaedica, 90, 297. 31084234 10.1080/17453674.2019.1612624 PMC6718169

[jsap70094-bib-0025] Salavati Schmitz, S. , Gow, A. , Bommer, N. , Morrison, L. & Mellanby, R. (2019) Diagnostic features, treatment, and outcome of dogs with inflammatory protein‐losing enteropathy. Journal of Veterinary Internal Medicine, 33, 2005–2013. 31381203 10.1111/jvim.15571 PMC6766500

[jsap70094-bib-0026] Simmerson, S.M. , Armstrong, P.J. , Wunschmann, A. , Jessen, C.R. , Crews, L.J. & Washabau, R.J. (2014) Clinical features, intestinal histopathology, and outcome in protein‐losing enteropathy in Yorkshire Terrier dogs. Journal of Veterinary Internal Medicine, 28, 331–337. 24467282 10.1111/jvim.12291 PMC4857982

[jsap70094-bib-0027] Smit, R.A.J. , Trompet, S. , Dekkers, O.M. , Jukema, J.W. & le Cessie, S. (2019) Survival bias in mendelian randomization studies: a threat to causal inference. Epidemiology, 30, 813–816. 31373921 10.1097/EDE.0000000000001072 PMC6784762

[jsap70094-bib-0028] Swales, H. , Batchelor, D.J. , Bodnarova, T. , Kent, A. , Campbell, S. , Gow, A.G. et al. (2025) A multicenter observational study comparing survival of pugs and dogs of other breeds with protein‐losing enteropathy. Journal of Veterinary Internal Medicine, 39, e70100. 40375559 10.1111/jvim.70100 PMC12081830

[jsap70094-bib-0031] Wennogle, S.A. , Olver, C.S. & Shropshire, S.B. (2021) Coagulation status, fibrinolysis, and platelet dynamics in dogs with chronic inflammatory enteropathy. Journal of Veterinary Internal Medicine, 35, 892–901. 33665845 10.1111/jvim.16092 PMC7995439

[jsap70094-bib-0032] Whitehead, J. , Quimby, J. & Bayliss, D. (2015) Seizures associated with hypocalcemia in a Yorkshire Terrier with protein‐losing enteropathy. Journal of the American Animal Hospital Association, 51, 380–384. 26535456 10.5326/JAAHA-MS-6205

[jsap70094-bib-0033] Wootton, F.E. , Hoey, C. , Woods, G. , Schmitz, S.S. , Reeve, J. , Larsen, J. et al. (2023) An undernutrition screening score for dogs with protein‐losing enteropathy: a prospective multicenter study. Journal of Veterinary Internal Medicine, 37, 1821–1829. 37480212 10.1111/jvim.16794 PMC10472980

